# The longitudinal age and birth cohort trends of regular exercise among adults aged 16–63 years in Sweden: a 24-year follow-up study

**DOI:** 10.1186/s12963-015-0049-x

**Published:** 2015-07-29

**Authors:** Matti Leijon, Patrik Midlöv, Jan Sundquist, Kristina Sundquist, Sven-Erik Johansson

**Affiliations:** Department of Clinical Science, Center for Primary Health Care Research, Lund University, Skåne University Hospital, Jan Waldenströms gata 35, 205 02 Malmö, Sweden; Stanford Prevention Research Center, Stanford University, Palo Alto, CA USA

**Keywords:** Exercise, Physical activity, Lifestyle, Longitudinal studies, Cohort effect, Mixed models, Public health

## Abstract

**Background:**

The aim of this study is to analyze longitudinally, based on four measurements at intervals of eight years, the annual effect of age group and birth cohort on regular exercise in the Swedish population from 1980–1981 to 2004–2005.

**Methods:**

We followed a randomly drawn subsample of individuals aged 16–63 years, interviewed by professional interviewers, from the Swedish Annual Level of Living Survey. We applied a mixed model with a random intercept and a random slope in order to analyze the annual effects.

**Results:**

The prevalence of regular exercise increased annually by 0.3 % among men and 0.7 % among women. For every one-unit increase in BMI, the odds of regular physical activity decreased by 6 % among men and 5 % among women. While the female birth cohorts all increased over time the male birth cohorts showed a different pattern, as only the three oldest birth cohorts (1926–1933, 1934–1941, 1942–1949) showed an increase in regular exercise. The three youngest birth cohorts (1958–1965, 1966–1973, 1974–1981) instead showed a decreased prevalence of regular exercise. There was an inverse relationship between regular exercise and age, although the differences between age groups tended to decrease over time. Differences related to educational level increased over time as the prevalence of exercise among those with higher educational attainment increased more than among those with lower educational attainment. The most dramatic relative increase in exercise over time (almost two-fold) was found among those who were obese or who reported a poor health status.

**Conclusions:**

The prevalence of regular exercise increased in all studied sub-groups. However, the increased difference related to education level is worrying. To reduce the risk for ill health in these groups, there is a need for targeted interventions.

## Background

Physical inactivity is the fourth-leading cause of chronic disease and mortality such as heart disease, stroke, diabetes, and cancers, contributing to over 3 million preventable deaths annually worldwide [1]. In spite of this, approximately 60 % of the adult population in the world does not reach the recommended level of physical activity [2]. According to statistics from WHO’s Global Burden of Disease project, physical inactivity is the fifth-leading risk factor for burden of disease for women in Sweden and the sixth-leading risk factor for men [3]. Danish data suggest that life expectancy is 5.3 years longer for a physically active 25-year-old man compared to a physically inactive man of the same age. The corresponding number for females is 5.1 years [4]. The health benefits of physical activity among adults include improved cardiorespiratory and muscular fitness, weight loss, reduced abdominal obesity, improved maintenance of weight loss, increased bone density, prevention of falls, reduced depression, and improved sleep quality [5].

Physical activity, exercise, and related terms have been defined somewhat inconsistently over the last decade [5–7]. The terms exercise and physical activity are often used interchangeably. However, nowadays exercise is seen as a subcategory of physical activity that is defined as “*planned, structured, and repetitive and purposive in the sense that the improvement or maintenance of one or more components of physical fitness is the objective,”* and generally refers to physical activity performed during leisure time [5].

A broad consensus has emerged in recent years in relation to the desirability of promoting physical activity and exercise, and support for increased physical activity is expressed in numerous policy documents from Sweden and elsewhere [5, 8–10]. Monitoring trends in populations is important from a public health perspective, with data from trend analyses making it possible to identify and target certain groups in the population when planning interventions.

Large Swedish studies from the late 1990s [11, 12] using a retrospective recall design indicate negative trends in physical activity in adults according to both age and calendar year. More recent studies presenting longitudinal exercise trends in adults showed a positive trend in physical activity over time [13–20]. A Danish study describing the time trend of leisure-time physical activity from 1987 to 2005 found an increase in leisure-time physical activity in the population that was mainly explained by a higher proportion of highly physically active individuals in more recent generations [13]. An Australian study of leisure-time physical activity, also including the time trends in relation to year of birth, age at the time of the interview, and year of interview [16], concluded that activity levels were independently influenced by age and time period, and in males, birth cohort as well. A Finnish study of trends in physical activity in relation to age, calendar year (1972–1997), and birth cohort in adults also found an increase in leisure-time physical activity, while the prevalence of occupational and commuting physical activity decreased [14]. The Eurobarometer study [21] and a more recent study by Bergman et al. [22] confirm reaching recommended physical activity levels is inversely related to age. A recent Swedish study showed an increase in physical activity from 1990 to 2007, with high increases among individuals in the higher educational group, especially after 1999. Furthermore, the gap between educational groups widened at the end of the study period [17]. The Danish study, on the other hand, showed that the inequality gap in activity level remained stable during the 18-year period [13].

In this longitudinal study we used data from four different measurements on the same individuals, in intervals of eight years, between 1980–1981 and 2004–2005, including all who had reported at least once, completed with new individuals in the age span 16–23 years. The aim of this study is to analyze longitudinally, based on four measurements on the same individuals, *at intervals of eight years,* the annual effect of age group and birth cohort on regular exercise in the Swedish population during a 24-year period, for each sex separately. Another aim is to analyze whether these effects remain after adjustment for possible confounders such as education, BMI, smoking, and self-reported health status.

## Methods

### The Swedish Annual Level of Living Survey

The Swedish Annual Level of Living Survey (SALLS), which has been conducted annually since 1974 by Statistics Sweden, was used as the source of data in this study [23]. The SALLS is a random sample of adult, non-institutionalized persons aged 16–84 years from the Total Population Register that represents the entire population of Sweden. Professional interviewers from Statistics Sweden conduct the interviews face-to-face, usually at the respondents’ homes [23]. The data are not publicly available, and the use and analysis of the data require permission from Statistics Sweden.

In this study, we used a longitudinal part of the 1980–1981, 1988–1989, 1996–1997 and 2004–2005 samples, including all individuals who had answered at least once, completed with new individuals in the age span 16–23 years. In this study, individuals aged between 16 and 63 years were included. Participants who were missing on one occasion were only excluded from that occasion (non-response). The non-response rate for the different surveys varied between 20 and 25 %. There were only minor differences in response rates between the longitudinal analysis and cross-sectional parts of the study. However, the response patterns for all four surveys were similar with regard to sex, age, place of living, and salary. By response pattern, it is meant that a certain level of a variable differs in response rate between another level in a non-random way over the years, e.g., women had higher response rates than men in all surveys. It is advantageous for longitudinal analyses that this pattern doesn’t change over the years, even if the non-response rate increases.

### Outcome variable

For the 1980–1981, 1988–1989 and 1996–1997 surveys, the outcome variable regular exercise was dichotomized as (0) never or now and then and (1) regularly (more than once a week) based on the response to the following question: How much do you exercise in your leisure time? Respondents selected the most appropriate of the following five answers: (1) I get practically no exercise at all; (2) I exercise occasionally (e.g., 1-h walks, skiing a couple of times every year, swimming, picking mushrooms); (3) I exercise regularly about once a week (e.g., fast walks, skiing, swimming, jogging, cycling); (4) I exercise regularly about twice a week (e.g., fast walks, skiing, swimming, jogging, cycling); and (5) I exercise regularly and vigorously at least twice a week (e.g., skiing, swimming, running, cycling, ball games). In the 2004–2005 survey, the following question was used: How much do you exercise in your leisure time? The possible answers were: (1) I get practically no exercise at all; (2) I exercise occasionally (for example, short walks); (3) I exercise regularly about once a week; (4) I exercise regularly about twice a week; and (5) I exercise regularly and vigorously at least twice a week. The following factors have, in previous studies, based on the same survey question, shown association with exercise: sex, education level, BMI, smoking habits, and self-reported health status [24, 25].

### Explanatory variables

In addition to the three time-related categories – survey period, age at the time of the interview, and birth cohort (based on year of birth) – we chose to include in the regression-based analysis the following explanatory variables: sex, education level, BMI, smoking status, and self-reported health status. All included variables were reported at each interview.

*Survey period* comprised four categories: 1980–1981, 1988–1989, 1996–1997 and 2004–2005. All individuals who answered the survey at least once were included.

*Age* at the time of interview was assessed to measure time trends and was categorized into the following groups: 16–23, 24–31, 32–39, 40–47, 48–55, and 56–63 years. The eight-year age categories reflect the eight-year intervals between surveys. In Tables 3 and 4, age is centered on 44 years.

*Birth cohort* (based on year of birth) was assessed to measure cohort effect and comprised the following groups: 1918–1925, 1926–1933, 1934–1941, 1942–1949, 1950–1957, 1958–1965, 1966–1973, 1974–1981 and 1982–1989. In Tables 3 and 4, cohort is centered on the birth year 1954.

*Sex*: separate analyses were undertaken for men and women.

*Educational level* was dichotomized as follows: (1) low-middle, compulsory school or less (≤9 years) or practical high school (vocational school) (10–11 years); and (0) high, theoretical high school and/or college (≥12 years).

*Body mass index (BMI),* calculated as weight (kg)/height^2^ (m^2^), was included as a continuous variable centered around 24 and 23 kg/m^2^ for men and women, respectively, and categorized as normal BMI (<25), overweight (25–30), and obese (>30). Weight and height were self-reported. Individuals with missing values for either weight or height were excluded from the dataset.

*Smoking:* respondents were divided into three groups: (1) never smokers; (2) former smokers (regardless of when they quit); and (3) daily smokers.

*Self-reported health status* was dichotomized as (1) good (those who described their health as good (1980–1981 and 1988–1989) or good or very good (1996–1997 and 2004–2005)) and (0) poor (all other answers).

### Statistical analysis

In the analysis, descriptive statistics were used to present the distributions of the explanatory variables (Table 1), as well as the prevalence of exercise in the different explanatory variable (Table 2) categories according to sex and survey period. We used mixed models to account for the repeated measurements.

**Table 1:** Distribution (%) of the different variables according to sex and survey year

Variable	Men				Women			
	1980/81	1988/89	1996/97	2004/05	1980/81	1988/89	1996/97	2004/05
**N**	2,448	2,363	2,344	1,943	2,418	2,338	2,337	1,971
Age (years)								
16-23	18.1	18.6	14.1	14.3	18.6	18.3	13.6	14.7
24-31	19.7	17.7	18.1	14.7	18.2	18.0	18.6	14.6
32-39	21.9	18.3	18.1	18.6	19.5	18.0	18.7	18.2
40-47	14.7	20.6	17.7	17.0	14.0	19.3	18.1	18.0
48-55	11.9	14.1	19.7	16.3	14.8	12.6	18.6	17.2
56-63	13.8	10.8	12.4	19.2	14.9	13.9	12.4	17.3
Education								
Low-middle	67.5	62.6	54.6	45.1	78.0	68.1	56.0	42.3
High	32.5	37.4	45.4	54.9	22.0	31.9	44.0	57.7
BMI								
Normal	68.4	65.3	55.6	48.8	79.0	76.9	69.5	66.1
Overweight	27.6	29.9	37.8	41.4	17.4	18.5	24.5	24.9
Obese	4.0	4.8	6.6	9.8	3.7	4.6	6.0	9.0
Smoking								
Never	39.2	44.8	48.7	54.2	49.2	49.5	49.4	51.4
Former	25.8	27.3	32.7	32.0	17.2	20.2	25.3	29.4
Daily	35.0	27.9	18.6	13.8	33.6	30.3	25.3	19.2
Self-reported health status								
Good	83.3	84.6	84.4	82.6	80.3	80.4	81.3	77.1
Poor	16.7	15.4	15.6	17.4	19.7	19.6	18.7	22.9

**Table 2:** Unadjusted prevalence of regular exercise in the different variable categories according to sex and survey year

Variable	Men				Women			
	1980/81	1988/89	1996/97	2004/05	1980/81	1988/89	1996/97	2004/05
n	2,448	2,363	2,344	1,943	2,418	2,338	2,337	1,971
Totals	32.9	35.5	39.2	44.7	26.0	29.6	36.7	48.3
Age (years)								
16-23	50.7^6)^	50.9^7)^	56.1^8)^	55.3^9)^	36.5^6)^	39.1^7)^	51.1^8)^	53.1^9)^
24-31	35.6^5)^	39.4^6)^	49.3^7)^	53.5^8)^	27.0^5)^	31.0^6)^	39.7^7)^	54.4^8)^
32-39	31.3^4)^	32.9^5)^	36.3^6)^	41.9^7)^	22.2^4)^	27.0^5)^	34.9^6)^	44.0^7)^
40-47	27.4^3)^	28.6^4)^	34.5^5)^	40.3^6)^	27.2^3)^	26.7^4)^	35.4^5)^	45.5^6)^
48-55	23.9^2)^	31.0^3)^	29.6^4)^	39.3^5)^	23.3^2)^	30.5^3)^	34.6^4)^	47.8^5)^
56-63	21.7^1)^	25.4^2)^	31.4^3)^	41.1^4)^	18.1^1)^	21.5^2)^	24.5^3)^	46.9^4)^
Education								
Low-middle	29.7	33.4	34.5	37.9	24.6	27.5	35.1	42.6
High	39.5	38.8	44.8	50.3	30.9	33.8	38.9	52.5
BMI								
Normal	36.9	40.3	44.0	50.5	27.1	31.8	41.1	51.5
Overweight	25.5	28.0	35.7	41.3	23.3	23.0	29.0	47.1
Obese	16.3	15.9	18.2	30.0	15.9	17.9	17.3	28.1
Smoking								
Never	41.7	44.0	47.5	52.6	28.2	33.9	42.1	53.8
Former	34.4	36.6	34.4	39.5	31.5	28.7	35.8	47.8
Daily	21.9	20.7	25.6	25.6	20.0	23.1	27.2	34.4
Self-reported health status								
Good	36.1	38.2	42.4	47.4	28.3	32.0	40.1	52.1
Poor	16.9	20.0	21.9	31.8	16.6	19.5	22.1	35.3

Cohort: ^1)^1918-1925; ^2)^1926-1933; ^3)^1934-1941; ^4)^1942-1949; ^5)^1950-1957; ^6)^1958-1965; ^7)^1966-1973;^8)^ 1974-1981;^9)^ 1982-1989

Two mixed logistic models with random intercepts and random slopes were applied to test the change in regular exercise over time according to age group and cohort. Model I included age, cohort, and the interaction age-by-cohort, age-squared, and cohort-squared (women only); Model II was also adjusted for all explanatory variables (Tables 3 and 4). The effects of the survey period do not need to be estimated for a longitudinal panel study as age and time are the same variable. Instead, the focus can be on the age-by-cohort interaction. Estimations of prevalence of regular exercise and prevalence odds ratio of regular exercise, both as a function of age and birth cohort and adjusted for other predictors, were obtained from a mixed logistic regression model. Based on predicted values from the adjusted mixed model, (Model II), estimates of percentage change in prevalence of Regular Exercise as a function of age and Birth Cohort were estimated by applying a linear regression (Tables 5 and 6). The statistical analyses were performed using the STATA software package version 12.

**Table 3:** Odds ratios (ORs with 95 % CIs) for regular exercise in men aged 16–63 years, obtained by applying mixed models with random intercepts and random slopes

		Model I*		Model II**	
Variable	Category	OR	95 % CI	OR	95 % CI
Fixed effects					
Rate of change					
Agec (Int)	Centered at 44 y	1.014	1.004-1.025	1.019	1.008-1.030
Agec*cohort		1.0007	1.0002-1.0013	1.0010	1.0004-1.0016
Agec-squared		1.0017	1.0011-1.0023	1.0018	1.0012-1.0024
Initial status					
Cohortc	Centered at 1954	1.04	1.03-1.05	1.03	1.02-1.04
Education	Low-Middle			1	
	High			1.53	1.33-1.76
Smoking	Non-smoker			1	
	Former			0.77	0.66-0.91
	Daily			0.32	0.27-0.39
BMI (kg/m^2^)	Centered at 24			0.94	0.92-0.96
Self-reported health status	Good			2.04	1.69-2.47
	Poor			1	
Random effects (unstructured)		Variance	Standard error	Variance	Standard error
Var (constant)		2.24	0.22	1.94	0.20
Var (agec)		0.0029	0.0010	0.0027	0.0010
Cov (agec; constant)		0.015	0.108	0.019	0.0098

*Adjusted for age, cohort, age-by-cohort interaction, and age-squared

**Adjusted also for educational level, smoking, BMI, and self-reported health status

**Table 4:** Odds ratios (ORs with 95 % CIs) for regular exercise in women aged 16–63 years, obtained by applying mixed models with random intercepts and random slopes

		Model I*		Model II**	
Variable	Category	OR	95 % CI	OR	95 % CI
Fixed effects					
Rate of change					
Agec (Int)	Centered at 44 y	1.06	1.05-1.07	1.07	1.06-1.08
Agec*cohort		1.003	1.002-1.005	1.003	1.002-1.005
Agec-squared		1.002	1.001-1.003	1.002	1.001-1.003
Initial status					
Cohortc	Centered at 1954	1.07	1.06-1.09	1.07	1.06-1.08
Cohortc-squared		1.0015	1.0007-1.0023	1.0015	1.0007-1.0023
Education	Low-Middle			1	
	High			1.14	1.01-1.30
Smoking	Non-smoker			1	
	Former			0.90	0.78-1.04
	Daily			0.53	0.46-0.61
BMI (Kg/m^2^)	Centered at 23			0.95	0.93-0.96
Self-reported health status	Good			1.76	1.52-2.05
	Poor			1	
Random effects (unstructured)		Variance	Standard error	Variance	Standard error
Var (constant)		1.01	0.12	0.87	0.11
Var (agec)		0.00067	0.00069	0.00020	0.00064
Cov (agec; constant)		−0.0085	0.0062	−0.011	0.0056

*Adjusted for age, cohort and age-by-cohort interaction, and age-squared

**Adjusted also for educational level, smoking, BMI and self-reported health status

**Table 5:** Prevalence (%) of regular exercise and annual change in prevalence of exercise (Δ% per year) in men aged 16–63 years according to age and birth cohort by Model II in Table 3

Variable			Age				
Birth cohort	16-23	24-31	32-39	40-47	48-55	56-63	Δ% cohort; p
1918-1925	-	-	-	-	-	21.7	-
1926-1933	-	-	-	-	22.6	27.1	0.56 **
1934-1941	-	-	-	25.8	28.6	34.1	0.52 ***
1942-1949	-	-	29.8	29.0	32.2	39.4	0.38 ***
1950-1957	-	37.9	33.4	33.6	38.3	-	−0.02 ns
1958-1965	49.1	41.5	38.4	39.1	-	-	−0.43 ***
1966-1973	52.4	46.3	43.4	-	-	-	−0.57 ***
1974-1981	55.3	52.1	-	-	-	-	−0.41 *
1982-1989	55.7	-	-	-	-	-	-
Δ% age	0.29	0.58	0.57	0.55	0.63	0.75	
p	***	***	***	***	***	***	

*p < 0.05; **p < 0.01; *** *p* < 0.001; ns = non-significant

1980/81 1988/89 1996/97 2004/05

**Table 6:** Prevalence (%) of exercise and annual change in prevalence of exercise (Δ% per year) in women aged 16–63 years according to age and birth cohort, predicted using Model II in Table 4

Variable			Age				
Birth cohort	16-23	24-31	32-39	40-47	48-55	56-63	Δ% cohort
1918-1925	-	-	-	-	-	19.2	
1926-1933	-	-	-	-	20.5	23.4	0.37***
1934-1941	-	-	-	22.9	25.1	32.1	0.56***
1942-1949	-	-	25.6	25.9	31.9	45.7	0.79***
1950-1957	-	29.8	29.0	33.7	45.5	-	0.62***
1958-1965	35.1	32.8	36.3	44.7	-	-	0.38***
1966-1973	38.4	40.2	47.0	-	-	-	0.53***
1974-1981	46.8	53.9	-	-	-	-	0.89***
1982-1989	55.5	-	-	-	-	-	-
Δ% age	0.84	0.95	0.87	0.92	1.00	1.10	
p	***	***	***	***	***	***	

*p < 0.05; **p < 0.01; ***p < 0.001

1980/81 1988/89 1996/97 2004/05

### Ethics

This study was approved by the ethical committee in Stockholm (approval no. 12/2000). There is no demand for written consent in this survey. All included individuals had the opportunity to deny participation, and they were informed about the use of data for research and that results will be reported only at the group level.

## Results

Table 1 shows the distribution of different explanatory variables – age, educational level, BMI, smoking, and self-reported health status – by sex and survey period. The overall pattern over time is of increases in educational attainment and BMI, a decline in the number of daily smokers, and somewhat decreased self-reported health.

In Table 2 the unadjusted prevalence (%) of regular exercise for the different variables is shown according to sex and survey period. Overall there was a trend toward increased regular exercise over the 24-year period in all age groups and for both men and women. Women showed a larger increase in exercise over time, with lower prevalence of regular exercise than males in all age groups in the first assessment period, but higher prevalence in all age groups, except for the 16- to 23-year age group, in the last assessment period. The largest increases in regular exercise prevalence among females were in those aged 48 years and older and those aged 24–31 years. The overall longitudinal trend shows that the increase was weaker during the 1980s and much stronger at the beginning of the new century. The results also show an inverse relationship between regular exercise and age, though the difference between the different age groups tended to decrease over time. The birth cohort effect shows differences according to sex. The prevalence of regular exercise increased over time among the 1942–1949, 1950–1957, 1958–1965, 1966–1973 and 1974–1981 female cohorts. Among men, the pattern was somewhat different, with the prevalence of regular exercise decreasing among men in the 1966–1973 and 1974–1981 cohorts, falling and then rising in the 1942–1949, 1950–1957 and 1958–1965 cohorts, and increasing in the 1934–1941 and 1926–1933 cohorts. Exercise increased with level of education in both men and women, and the differences in regular exercise between those with lower and higher educational attainment increased over time. Exercise increased among non-smokers, former smokers, and daily smokers, with a pattern quite similar to the overall pattern. The most dramatic relative increase in regular exercise was found among those who were obese or who reported a poor health status. In these two groups, there was an almost two-fold increase in exercise over time.

Tables 3 and 4 show the ORs for regular exercise in two mixed models with random intercepts and random slopes. Model I included age (centered), birth cohort (centered), the interaction between age and birth cohort, age-squared, and cohort-squared (women only); Model II was also adjusted for educational level, BMI (centered around its mean), smoking, and health status. The adjusted model (Model II) shows similar odds ratios for age (significant for women), birth cohort (significant for men and women) and the age-by-cohort interaction (significant for men and women) as the initial model (Model I). According to Model II, the birth cohort effect (centered at 1954) shows an average annual increase in the prevalence of regular exercise of 0.3 % for men and 0.7 % for women.

Both men and women with higher educational attainment had higher odds of regular exercise than those with lower educational attainment. The differences were greater among men (OR = 1.53, 95 % CI = 1.33–1.76) than among women (OR = 1.14, 95 % CI = 1.01–1.3). The odds of exercise were, as expected, considerably higher among those with good self-reported health than among those with poor self-reported health (OR = 2.04, 95 % CI = 1.69–2.47 among males; OR = 1.76, 95 % CI = 1.52–2.05 among females). BMI was also closely related to regular exercise: for every one-unit increase in BMI, the odds of regular physical activity decreased by 6 % among men (OR = 0.94, 95 % CI = 0.92—0.96) and 5 % among women (OR = 0.95, 95 % CI = 0.93–0.96).

The prevalence of regular exercise and annual change in regular exercise, predicted based on Model II in Tables 3 and 4, are presented according to age group, cohort (birth year), and assessment period in Tables 5 and 6. By applying a linear regression model, the annual changes in age groups and birth cohorts were estimated. During the study period, there was a mean annual increase in regular exercise ranging from 0.38 to 0.56 % in the three birth cohorts between 1926 and 1949. There were no significant changes in the 1950–1957 birth cohort. There was an average annual decrease in regular exercise of 0.41 to 0.57 % in the three birth cohorts between 1958 and 1989. Among women, regular exercise increased in all birth cohorts, with average annual changes for individual birth cohorts ranging from 0.37 to 0.89 %. Analysis of the age effect showed significant annual increases in all age groups, which varied between 0.29 and 0.75 % among men and between 0.84 and 1.10 % among women.

## Discussion

During a 24-year period, from 1980–1981 to 2004–2005, the prevalence of regular exercise increased in all studied sub-groups among both men and women. Data also show an inverse relation to age, even though the differences between age groups decreased over time. Women had a higher annual increase in regular exercise than men over the 24-year study period, especially among those aged 48 years and older. Differences related to educational level increased over time as the prevalence of those with higher educational attainment increased more than the prevalence of those with lower educational attainment.

According to the adjusted model, the prevalence of regular exercise increased annually by 0.3 % among men and 0.7 % among women. While the female birth cohorts all increased over time, the male birth cohorts showed a different pattern, as only the three oldest birth cohorts (1926–1933, 1934–1941, 1942–1949) showed an increase in regular exercise. The three youngest birth cohorts (1958–1965, 1966–1973, 1974–1981) instead showed a decreased prevalence of regular exercise.

Our findings were in agreement with another Swedish study [17] that showed an increase in physical activity (1990–2007) with higher increases among those in the higher educational group, especially after 1999. Our data support that this trend has been going on for a longer period than that. Moreover, our data support the conclusion from a Swedish study (VIP) where the gap between educational groups also widened in more recent years [17]. This was not the case in the Danish study, showing that the inequality gap in activity level remained stable during from 1987 to 2005 [13].

The most dramatic relative increase in exercise over time was found among those who were obese or who reported a poor health status. BMI is closely related to calorie intake and energy expenditure related to physical activity and exercise. This longitudinal study showed that for every one-unit increase in BMI the odds of regular physical activity decreased by 6 % among men and 5 % among women.

The overall finding of increase in regular exercise over time and the inverse relationship between age and regular exercise is consistent with previous Swedish and international findings [11–16, 18–20]. Few studies so far have included analyses of both age and cohort effect on regular exercise [13].

We have no data on the prevalence of occupational and commuting physical activity like in the Finnish study [14]. Hence, the same pattern, including lower levels of occupational and commuting physical activity together with an increase in leisure-time physical activity, is also highly likely for Sweden during a similar time period. These changes are certainly due to changes in society and in the labor market, including more sedentary time at work and fewer jobs that include physical labor.

### Limitations and strengths

This study has some important limitations that should be balanced against its strengths. One important limitation is that our outcome measures are based on self-reported assessments. Multiple studies have shown that self-reporting tools for assessment of physical activity are accurate and reliable when compared to objective quantification by activity monitoring or directly measured energy expenditure [26]. As with all self-reported information, there is always a risk of recall or social desirability bias when using self-reported physical activity as an outcome measure [27]. Such biases should be kept in mind when interpreting the results of studies using self-reported information.

However, the level of self-report bias is probably the same for all four time periods, thus most likely resulting in correct estimates of change between the periods. It is possible, though, that the bias of overestimation caused by self-report is not the same for all assessments, since public awareness of the importance of physical activity may have increased during the study period. This awareness can also be different between groups and result in an even higher social desirability bias among females and more educated people. Another limitation is that the non-response rate may have resulted in an overestimation of activity level, as those inactive could have been overrepresented among non-responders. On the other hand, the non-response rate in this study was relatively low (20–25 %) compared to surveys from other countries and was similar during the four assessment periods and similar in different sex, age, and region subgroups. The change in wording in the survey question (in 2004–2005 the examples were excluded) must also be considered as an important shortcoming. However, the dichotomization of the question is based on the keyword regular, making this shortcoming a little less important.

This study also has several strengths. One key strength is the follow-up of regular physical activity among individuals for a relatively long period of time (24 years). Together with this follow-up time, the repeated measurements make this study unique. A second strength is that the SALLS is one of the most comprehensive national surveys to date and has been conducted in Sweden for more than 30 years. The sample size is large and, unlike many surveys, each SALLS represent a simple random sample with a longitudinal “panel” with repeated measurements, drawn from the Total Population Register, and is thus representative of the entire Swedish population. Another advantage is that period effect can be excluded and substituted by the interaction between age and birth cohort. The surveys in the present study, mainly conducted at the respondents’ homes as face-to-face interviews by well-trained interviewers, have low non-response rates (about 24 %) with few missing data. The reliability of the survey questions has been estimated by re-interviewing a sample of the participants (test-retest method). The kappa coefficients were 0.64 for self-rated health and 0.58 for physical activity [28].

## Conclusions

The main results in this study are positive from a public health point of view, as the prevalence of regular exercise increased over time. Nevertheless, as the overall aim of public health work in Sweden and elsewhere is to decrease differences in health between groups, the trend of increased differences in regular exercise according to education level is worrying. Another possible threat is the negative trend in regular exercise in some of the middle-aged male cohorts. To reduce the risk for ill health in these groups there is a need for targeted interventions.
